# Modified generalized method of moments for a robust estimation of polytomous logistic model

**DOI:** 10.7717/peerj.467

**Published:** 2014-07-01

**Authors:** Xiaoshan Wang

**Affiliations:** Department of Clinical and Translational Research/Forsyth Institute, Cambridge, MA, USA; Department of Oral Health Policy and Epidemiology, Harvard School of Dental Medicine, Cambridge, MA, USA

**Keywords:** Robust statistics, Generalized method of weighted moments, Polytomous logistic model

## Abstract

The maximum likelihood estimation (MLE) method, typically used for polytomous logistic regression, is prone to bias due to both misclassification in outcome and contamination in the design matrix. Hence, robust estimators are needed. In this study, we propose such a method for nominal response data with continuous covariates. A generalized method of weighted moments (GMWM) approach is developed for dealing with contaminated polytomous response data. In this approach, distances are calculated based on individual sample moments. And Huber weights are applied to those observations with large distances. Mellow-type weights are also used to downplay leverage points. We describe theoretical properties of the proposed approach. Simulations suggest that the GMWM performs very well in correcting contamination-caused biases. An empirical application of the GMWM estimator on data from a survey demonstrates its usefulness.

## Introduction

Polytomous logistic regression models for multinomial data are a powerful technique for relating dependent categorical responses to both categorical and continuous explanatory covariates (McCullagh & Nelder, 1989; Liu & Agresti, 2005). In practice, however, the model building process can be highly influenced by peculiarities in the data. The maximum likelihood estimation (MLE) method, typically used for the polytomous logistic regression model (PLRM), is prone to bias due to both misclassification in outcome and contamination in the design matrix (Pregibon, 1982; Copas, 1988). Hence, robust estimators are needed.

For categorical covariates, we may apply MGP estimator (Victoria-Feser & Ronchetti, 1997), $\phi $-divergence estimator (Gupta et al., 2006), and robust quadratic distance estimator (Flores, 2001). The least quartile dfference estimator can deal with overdispersion problem (Mebane & Sekhon, 2004). But all these methods are difficult to adapt for continuous covariates.

A generalized method of moments (GMM) estimation can be formed as a substitute of MLE. The GMM is particularly useful when the moment conditions are relatively easy to obtain. GMM has been extensively studied in econometrics (Hansen, 1982; Newey & West, 1987; Pakes & Pollard, 1989; Hansen, Heaton & Yaron, 1996; Newey & McFadden, 1994). Under some regularity conditions, the GMM estimator is consistent (Hansen, 1982). With an appropriately chosen weight matrix, GMM achieves the same efficiency as the MLE (Hayashi, 2000). Furthermore, under certain circumstances, GMM provides more flexibility, such as dealing with endogeneity through instrumental variables (Baum, Schaffer & Stillman, 2002).

Like MLE, GMM estimation can be easily corrupted by aberrant observations (Ronchetti & Trojani, 2001). Such observations can bring up disastrous bias on standard parameter estimates if they are not properly accounted for, see Huber (1981), Hampel et al. (2005), and Rousseeuw & Leroy (2003). So we propose a modified estimation method based on an outlier robust variant of GMM. The method is different from the kernel-weighted GMM developed for linear time-series data by Kuersteiner (2012) in that this is a data-driven method for defining weights. The new approach is evaluated using asymptotic theory, simulations, and an empirical example.

**Figure 1:**
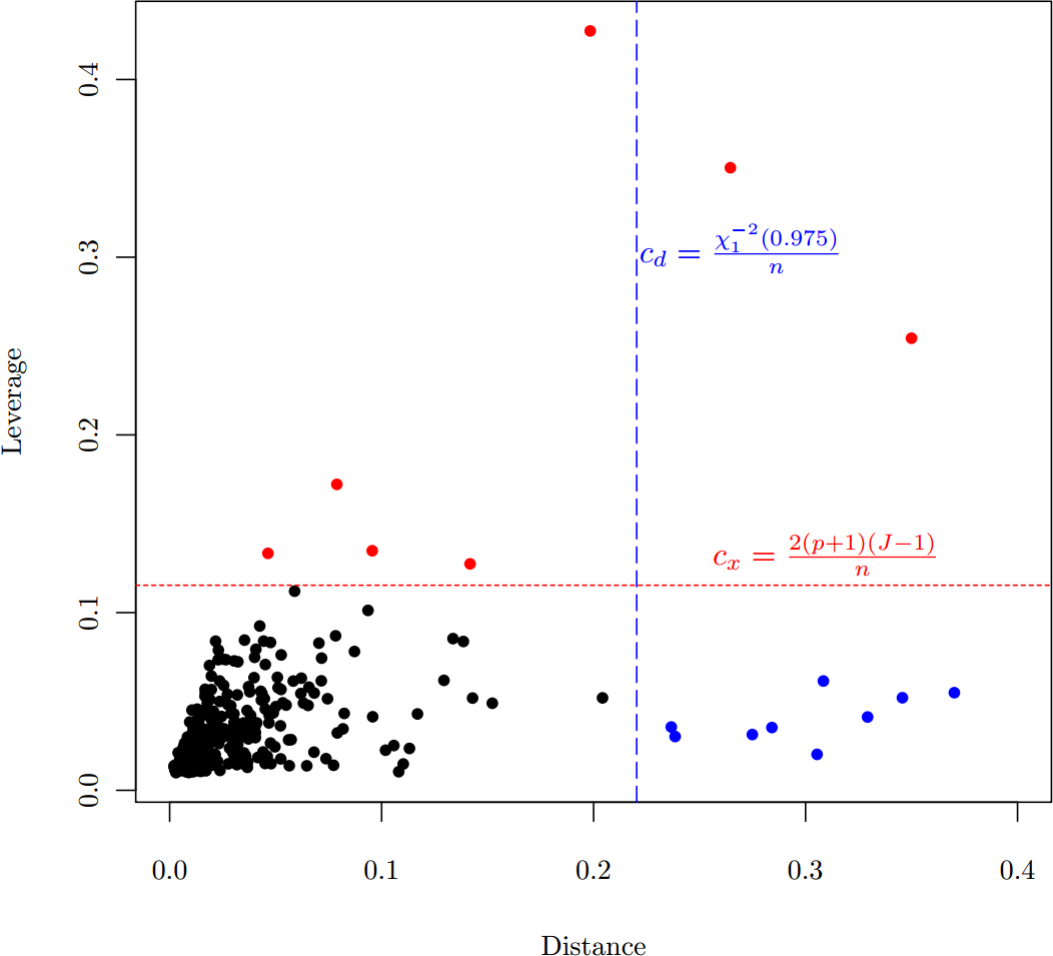
Scatter plot of distance vs. leverage, which are based on MLE. Criteria ${c}_{d}$ for the distance and ${c}_{x}$ for the leverage are demonstrated.

**Figure 2:**
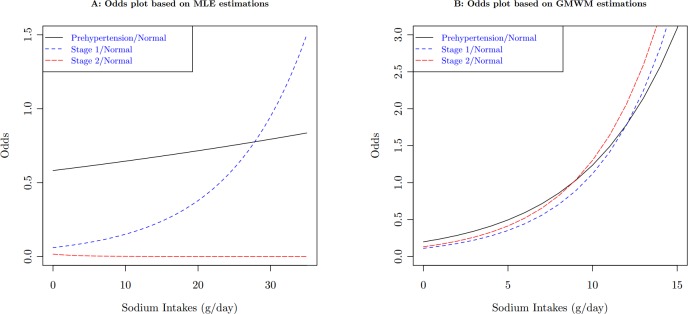
Compare odds plots of sodium intakes between MLE estimates and GMWM estimates on the population of female, age $=$ 40, and BMI $=$ 23.

The robust GMM estimator is motivated by the data from a 2006 study on hypertension in a sample of the Chinese population. 520 people completed the survey. Observed variables included demographics, social-economic status, weight, height, blood pressure, and food consumption. Sodium intakes were calculated based on overall food consumption. Among those covariates, age, body mass index (BMI), and sodium intakes are all continuous. Based on blood pressure measurements, subjects were classified into 4 categories: Normal, Pre-hypertension, Stage 1 and Stage 2 hypertension. Table 1 lists the summary statistics of the sample. One of the research objectives is to examine the association between hypertension and risk factors in the population. Since the proportional odds assumption is violated (Score test for the proportional odds assumption gives ${\chi }^{2}=182.27$ with a degree of freedom of 8, $p\lt 0.0001$), we apply the polytomous logistic model, using the normal category as the reference level. In the case of $J$ category, the polytomous logit model have $J-1$ comparisons. Each comparison have a set of parameters for all covariates in the model. Therefore, the generalized logit model is not parsimonious when comparing with the proportional odds model. But the simultaneous estimation of all parameters is more efficient than separate models for each comparison. It is another option for ordinal response data, especially when a proportional odds model does not fit the data well. Table 2 lists the output from the model estimated by MLE. It is obvious that, if MLE is used, the estimates is inconsistent for sodium intakes, particularly the negative coefficient of sodium intake for the odds between the Stage 2 hypertension and the Normal categories. The inconsistency is more obvious when we plot the odds with respect to the sodium intake, the downward trend of the odds in Fig. 2A. This result contradicts the previous finding that there is a strong relationship between sodium intake and hypertension, see for example National Research Council (2005), He & MacGregor (2004) and references therein. Besides, Fig. 2A also shows another strange situation: the higher starting points for the odds between the Pre-hypertension and the Normal categories. The scatter plot (Fig. 1) between distances and leverages suggests some observations are possible outliers: Observations 21, 33, 85, 92, 194, 274, 336, 414, 459, 483, and 489 have large distances, which are blue-colored, and Observations 37, 83, 263, 459, 483, 485, and 490 have large leverages, which are red-colored.

**Table 1:** Summary statistics for surveyed subjects.

Covariate		Hypertension categories			
		Normal	Pre-hypertension	Stage 1	Stage 2
Gender	Male	138	104	29	8
	Female	87	114	31	9
Age	Mean	43.2	48.8	54.3	60.3
	Std. Dev.	13.7	13.8	12.2	13.4
BMI	Mean	43.2	48.8	54.3	60.3
	Std. Dev.	13.7	13.8	12.2	13.4
Sodium intake	Mean	3.7	3.7	4.6	2.7
	Std. Dev.	3.0	2.4	5.0	2.1

**Table 2:** Polytomous logistic regression of a hypertension data: coefficient estimates and standard errors from GMWM and MLE.

Variable	Coefficients	MLE			GMWM		
		Estimates	Std. Err	$p$ value	Estimates	Std. Err	$p$ value
Sex	${\beta }_{21}$	0.7062	0.2022	0.0002	1.3339	0.2269	<0.0001
	${\beta }_{31}$	0.9789	0.3235	0.0012	1.0368	0.3013	0.0003
	${\beta }_{41}$	1.4193	0.5746	0.0068	0.6753	0.2195	0.0010
Age	${\beta }_{22}$	0.0350	0.0075	<0.0001	0.0671	0.0086	<0.0001
	${\beta }_{32}$	0.0715	0.0121	<0.0001	0.1139	0.0133	<0.0001
	${\beta }_{42}$	0.1096	0.0216	<0.0001	0.0753	0.0103	<0.0001
BMI	${\beta }_{23}$	0.1147	0.0316	0.0001	0.1681	0.0360	<0.0001
	${\beta }_{33}$	0.2422	0.0474	<0.0001	0.4382	0.0538	<0.0001
	${\beta }_{43}$	0.4351	0.0884	<0.0001	0.2279	0.0388	<0.0001
Sodium	${\beta }_{24}$	0.0104	0.0349	0.3829	0.1831	0.0355	<0.0001
	${\beta }_{34}$	0.0919	0.0426	0.0155	0.2315	0.0486	<0.0001
	${\beta }_{44}$	−0.2699	0.1580	0.9562	0.2294	0.0353	<0.0001

**Notes.**

Std. Err, standard error.

The paper is set up as follows. In the next section we presents the basic notations, model, and standard GMM. “A robust GMM” introduces the outlier robust GMM estimator, and gives a detailed exposition of its implementation. In “Results”, we compares the performance of the standard MLE with the new estimator using a Monte-Carlo experiment. And we apply both estimators to real epidemiological data, and illustrate the usefulness of the robust estimator for application oriented researchers. We conclude with a discussion of advantages and limitations of the approach. The supporting document gathers the proofs of the asymptotic property.

## Materials and Methods

### The baseline-category logit model

Assume a random sample of size $n$ from a large population. Each element in the population may be classified into one of $J$ categories, denoted by ${\mathbf{y}}_{i}=({y}_{i 1},{y}_{i 2},\ldots ,{y}_{i J})$ the multinomial trial for subject $i$, where ${y}_{i j}=1$ when the response is in category $j$ and ${y}_{i j}=0$ otherwise, $i=1,\ldots ,n$, $j=1,\ldots ,J$. Thus, $\sum _{j}{y}_{i j}=1$. Suppose $p$ explanatory covariates, with at least one of them being continuous, are observed. Define ${\mathbf{x}}_{i}=(1,{x}_{i 1},\ldots ,{x}_{i p})$, and $\mathbf{x}=({\mathbf{x}}_{1},\ldots ,{\mathbf{x}}_{n})$. We assume that $({\mathbf{y}}_{i},{\mathbf{x}}_{i})$ are independently and identically distributed ($i.i.d.$). Let ${\pi }_{i j}={\pi }_{j}({\mathbf{x}}_{i})=P({Y}_{i}=j\vert {\mathbf{x}}_{i})$, denote the probability that the observation of $Y$ belongs to category $j$, given covariates ${\mathbf{x}}_{i}$, we assume the relationship between the probability ${\pi }_{j}$ and $\mathbf{x}$ can be modeled as:

(1) \begin{eqnarray*} l o g\left\{\frac{{\pi }_{j}({\mathbf{x}}_{i})}{{\pi }_{J}({\mathbf{x}}_{i})}\right\}={\mathbf{x}}_{i}^{T}{\beta }_{j},\quad j=2,\ldots ,J \end{eqnarray*}

where ${\beta }_{j}^{T}=({\beta }_{j 0},{\beta }_{j 1},\ldots ,{\beta }_{j p})$. Here we set the first category as reference class. This model is called a baseline-category logit model (Agresti, 2012) or generalized logit model (Stokes, Davis & Koch, 2009). MLE is usually used for obtaining parameter estimation of this model. Here we present an alternative estimation method formed with the GMM.

### Estimation using GMM

The baseline-category logit model can be viewed as a multivariate model. Define ${\mathbf{y}}_{i}^{\ast T}=({y}_{i 2},\ldots ,{y}_{i J})$, since ${y}_{i 1}$ is redundant. Let ${\mathbf{X}}^{T}=({X}_{1}^{T},\ldots ,{X}_{n}^{T})$ is a $n(J-1)\times (p+1)(J-1)$ matrix, with ${X}_{i}^{T}$, a $(J-1)\times (p+1)(J-1)$ matrix, defined as:

(2) \begin{eqnarray*} {X}_{i}^{T}=\left(\begin{array}{@{}cccc@{}} \displaystyle {\mathbf{x}}_{i}^{T}&\displaystyle &\displaystyle &\displaystyle \\ \displaystyle &\displaystyle {\mathbf{x}}_{i}^{T}&\displaystyle &\displaystyle \\ \displaystyle &\displaystyle &\displaystyle \cdots &\displaystyle \\ \displaystyle &\displaystyle &\displaystyle &\displaystyle {\mathbf{x}}_{i}^{T} \end{array}\right). \end{eqnarray*}

In the GMM framework, we define

(3) \begin{eqnarray*} u(\boldsymbol{\beta})={X}_{i}({\mathbf{y}}_{i}^{\ast }-{\boldsymbol{\pi}}_{i}),\quad i=1,\ldots ,n \end{eqnarray*}

where ${\boldsymbol{\pi}}_{i}^{T}=({\pi }_{i 2},{\pi }_{i 3},\ldots ,{\pi }_{i J})$. And ${\boldsymbol{\beta}}^{T}=({\beta }_{2}^{T},{\beta }_{3}^{T},\ldots ,{\beta }_{J}^{T})$ is the $(p+1)(J-1)$ vector of unknown parameters. The population moment condition is

\begin{eqnarray*} E\{ u(\boldsymbol{\beta})\} =0, \end{eqnarray*}

with the corresponding sample moment condition

(4) \begin{eqnarray*} {U}_{n}(\boldsymbol{\beta})=\sum _{i=1}^{n}u(\boldsymbol{\beta}). \end{eqnarray*}

The GMM estimation of ${\hat {\boldsymbol{\beta}}}_{M}$ can be obtained by minimizing the following quadratic objective function

\begin{eqnarray*} {Q}_{n}(\boldsymbol{\beta})={U}_{n}^{T}(\boldsymbol{\beta}){\Sigma }_{n}^{-1}(\boldsymbol{\beta}){U}_{n}(\boldsymbol{\beta}), \end{eqnarray*}

where ${\Sigma }_{n}(\boldsymbol{\beta})$ can be the empirical variance–covariance matrix given by

\begin{eqnarray*} {\Sigma }_{n}(\boldsymbol{\beta})=\frac{1}{{n}^{2}}\sum _{i=1}^{n}{u}^{T}(\boldsymbol{\beta})u(\boldsymbol{\beta})-\frac{1}{n}{U}_{n}(\boldsymbol{\beta}){U}_{n}^{T}(\boldsymbol{\beta}). \end{eqnarray*}

Or, for the best efficiency of the GMM estimation, we can take the information matrix of the polytomous logit model (PLRM), that is,

(5) \begin{eqnarray*} {\Sigma }_{n}(\boldsymbol{\beta})=\sum _{i=1}^{n}{X}_{i}({D}_{i}-{\boldsymbol{\pi}}_{i}{\boldsymbol{\pi}}_{i}^{T}){X}_{i}^{T} \end{eqnarray*}

where ${D}_{i}=d i a g o n a l({\boldsymbol{\pi}}_{i}).$

In general, ${\hat {\boldsymbol{\beta}}}_{M}$ can be computed via an iterative procedure (Hansen, Heaton & Yaron, 1996). Under standard regularity conditions, the GMM estimator ${\hat {\boldsymbol{\beta}}}_{M}$ exists and converges in probability to the true parameter ${\boldsymbol{\beta}}_{0}$ (Hansen, 1982). A proof of asymptotic normality of GMM can be found on p. 2148 of Newey & McFadden (1994).

### A robust GMM

In this section we introduce the outlier robust GMM estimator. In the following subsection, we specify moment conditions used for robust estimation. And the details on the implementation of the estimator follows.

#### The generalized method of weighted moments

The main principle used in the robust GMM estimator is that we replace moment conditions by a set of observation weighted moment conditions. Instead of Eq. (3), we define

(6) \begin{eqnarray*} {u}^{w}(\boldsymbol{\beta})={w}_{i}{X}_{i}({\mathbf{y}}_{i}^{\ast }-{\boldsymbol{\pi}}_{i})-{c}_{i},\quad i=1,\ldots ,n \end{eqnarray*}

where ${c}_{i}=E\{ {w}_{i}{X}_{i}({\mathbf{y}}_{i}^{\ast }-{\boldsymbol{\pi}}_{i})\} $. Then the estimation can be based on the moment conditions

\begin{eqnarray*} E\{ {u}^{w}(\boldsymbol{\beta})\} =0. \end{eqnarray*}

Consequently, the generalized method of weighted moments (GMWM) estimates can be defined by

(7) \begin{eqnarray*} {\hat {\beta }}^{w}=\arg \min _{\beta \in \mathcal{B}}{Q}_{n}^{w}(\boldsymbol{\beta}) \end{eqnarray*}

where

(8) \begin{eqnarray*} {Q}_{n}^{w}(\boldsymbol{\beta})=[{U}_{n}^{w}(\boldsymbol{\beta})]^{T}\{ {\Sigma }_{n}^{w}(\boldsymbol{\beta})\} ^{-1}{U}_{n}^{w}(\boldsymbol{\beta}), \end{eqnarray*}

with

(9) \begin{eqnarray*} {U}_{n}^{w}(\boldsymbol{\beta})=\sum _{i=1}^{n}{u}^{w}(\boldsymbol{\beta}). \end{eqnarray*}

Here we take the summation as the sample moment condition. The advantage of using the summation is that it can lead us to a direct estimation of covariance matrix.

It is clear to see that this definition is analogous to the standard GMM. If we choose ${w}_{i}=1$ and ${c}_{i}=0$ for all observations, the moment conditions in (6) are reduced to the standard moment conditions. Therefore, the standard GMM is a special case of the GMWM.

In order to specify the weights for the robust GMM estimator, we need the following definition of a distance, which is based on individual moment conditions:

(10) \begin{eqnarray*} {d}_{i}(\boldsymbol{\beta})=[{u}_{i}^{w}(\boldsymbol{\beta})]^{T}\{ {\Sigma }_{n}^{w}(\boldsymbol{\beta})\} ^{-1}{u}_{i}^{w}(\boldsymbol{\beta}),\quad i=1,\ldots ,n. \end{eqnarray*}

The weight is assigned based on ${d}_{i}(\boldsymbol{\beta})$, that is, ${w}_{d}=w\left({d}_{i}(\boldsymbol{\beta})\right)$. There are several alternative specifications of weight functions available in the literature (Huber, 1981; Hampel et al., 2005). In this study, the Huber’s weights are applied:

(11) \begin{eqnarray*} w\left({d}_{i}(\boldsymbol{\beta})\right)=\min \left(1,\frac{{c}_{d}}{{d}_{i}(\boldsymbol{\beta})}\right). \end{eqnarray*}

The above specification of weight function requires a value of the tuning constant ${c}_{d}$. Both the outlier sensitivity and the efficiency of the estimator are determined by the constant. On the one hand, the estimator should be reasonably efficient if the sample contains no outlier. On the other hand, the estimator should be insensitive to outliers. To determine ${c}_{d}$, understanding the distribution of ${d}_{i}(\boldsymbol{\beta})$ is critical. Clearly, ${u}_{i}^{w}(\boldsymbol{\beta})$ is a column vector, and ${d}_{i}(\boldsymbol{\beta})$ is a scalar quadratic distance, so we set ${c}_{d}={\chi }_{1}^{-2}(0.975)/n$, where ${\chi }_{p}^{-2}(\cdot )$ is the quantile of the ${\chi }^{2}$ distribution with $p$ degrees of freedom.

If we take the information matrix (5) of the PLRM as ${\Sigma }_{n}^{w}(\boldsymbol{\beta})$, we can compute leverage for each observation:

(12) \begin{eqnarray*} {H}_{i}={X}_{i}\{ {\Sigma }_{n}^{w}(\boldsymbol{\beta})\} ^{-1}{X}_{i}^{T}{\sigma }_{i}^{w},\quad i=1,\ldots ,n \end{eqnarray*}

where ${\sigma }_{i}^{w}$ is the $i\mathrm{th}$ component of ${\Sigma }_{n}^{w}(\boldsymbol{\beta})$. Then, a Mallows-type weight can be defined based on $t r a c e({H}_{i})$; that is, ${w}_{x}=w(t r a c e({H}_{i}))$, to downplay the observations with high leverages. Lesaffre & Albert (1989) suggest that the practical rule for isolating leverage points might set ${c}_{x}=2(p+1)(J-1){{/}}n$. In this study, we give observations with large leverages 0 weights,

(13) \begin{eqnarray*} {w}_{x}=w(t r a c e({H}_{i}))=\left\{\begin{array}{@{}ll@{}} \displaystyle 1&\displaystyle \text{if }t r a c e({H}_{i})\leq \frac{2(p+1)(J-1)}{n}\\ \displaystyle 0&\displaystyle \text{otherwise. } \end{array}\right. \end{eqnarray*}

An approach often used to combine the two weights is ${w}_{i}={w}_{d}\cdot {w}_{x}$ (Heritier et al., 2009).

The consistency correction vector ${c}_{i}$ is defined as

\begin{eqnarray*} {c}_{i}=\left(w\left({d}_{i}^{(1)}(\boldsymbol{\beta})\right)-w\left({d}_{i}^{(0)}(\boldsymbol{\beta})\right)\right)/d i a g\left({\Sigma }_{n}^{w}(\boldsymbol{\beta})\right),\quad i=1,\ldots ,n \end{eqnarray*}

where $w\left({d}_{i}^{(h)}(\boldsymbol{\beta})\right)=w\left({X}_{i}\{ h-{\pi }_{i}(\boldsymbol{\beta})\} /d i a g{\left[{\Sigma }_{n}^{w}(\boldsymbol{\beta})\right]}^{-1}\right)$ with $h=\{ 0,1\} $, is the weight for ${y}_{i}^{\ast }$.

#### Implementation of the estimator

The continuous updating estimation method is applied in this study for estimating the regression coefficients and corresponding variance. The procedure is detailed as follows:

1. Apply an initial value ${\beta }^{(0)}$ for computing ${\Sigma }_{n}\left(\boldsymbol{\beta}\right)$.
2. Compute ${d}_{i}(\boldsymbol{\beta})$ using Eq. (10) and ${H}_{i}$ using Eq. (12); assign weights correspondingly based on (11) and (13).
3. With the combined weights, calculate ${\Sigma }_{n}^{w}(\boldsymbol{\beta})$ and ${U}_{n}^{w}(\boldsymbol{\beta})$ in Eq. (9).
4. Obtain the estimator ${\hat {\boldsymbol{\beta}}}_{w}^{(1)}$ by minimizing ${Q}_{n}^{w}$ of Eq. (8).
5. Go back to Step 1, replace ${\boldsymbol{\beta}}^{(0)}$ with the estimator ${\hat {\boldsymbol{\beta}}}_{w}^{(1)}$ in computing ${\Sigma }_{n}^{w}\left({\hat {\boldsymbol{\beta}}}_{w}^{(1)}\right)$, and move to the next iteration.
6. Continue this procedure until convergence criteria are met.

For the starting value ${\beta }^{(0)}$, a reasonable choice is the MLE estimation based on the original data.

In the appendix, we proved that, under some regularity assumptions, we can have that ${\hat {\boldsymbol{\beta}}}_{w}$ is consistent for ${\boldsymbol{\beta}}_{0}$. And by studying the behavior of the weighted moment equations in a neighborhood of ${\boldsymbol{\beta}}_{0}$, we showed that the asymptotic linearity ensures the applicability of the central limit theorem for the asymptotic normality of GMWM.

## Results

### Monte Carlo simulations

In this section we investigate the properties of the GMWM estimator using a Monte-Carlo study. We generate data with three response categories and two covariates which are from multivariate normal distribution with 0 mean and identity covariance. The true coefficient matrix ${\boldsymbol{\beta}}_{0}$ is

\begin{eqnarray*} {\boldsymbol{\beta}}_{0}=\left(\begin{array}{@{}ccc@{}} \displaystyle {\beta }_{10}&\displaystyle {\beta }_{20}&\displaystyle {\beta }_{30}\\ \displaystyle {\beta }_{11}&\displaystyle {\beta }_{21}&\displaystyle {\beta }_{31}\\ \displaystyle {\beta }_{12}&\displaystyle {\beta }_{22}&\displaystyle {\beta }_{32} \end{array}\right)=\left(\begin{array}{@{}crr@{}} \displaystyle 0&\displaystyle 1.0&\displaystyle -0.3\\ \displaystyle 0&\displaystyle -0.8&\displaystyle 0.7\\ \displaystyle 0&\displaystyle -1.0&\displaystyle -0.5 \end{array}\right). \end{eqnarray*}

Based on the specified coefficient values and using the probability based on the model (1), we compute the category-specific probabilities for each subject. Then, using the computed probabilities, we determine the most likely category to which each subject belongs. This decision is made through random generation from the multinomial distribution with the probability vector as a parameter. For instance, multinomial categories in R-Language are generated using $r m u l t i n o r m({n}_{i},{N}_{i},\pi ({\mathbf{x}}_{i}))$ function, where $\pi ({\mathbf{x}}_{i})=({\pi }_{1}({\mathbf{x}}_{i}),\ldots ,{\pi }_{J}({\mathbf{x}}_{i}))$ is the probability vector, ${n}_{i}$ is the number of random vectors to draw, and ${N}_{i}$ is the total number of objects that are put into $J$-categories. In our case, ${n}_{i}={N}_{i}=1$ for all subjects and $J=3$.

Two sample sizes, 100 and 1000, are examined. For each sample size, we run the simulation 1000 times. Average biases and MSEs are calculated and tabulated. Table 3 shows the results from randomly generated data with no outliers added. When the sample size is small, GMWM will give greater biases on ${\beta }_{30}$ and ${\beta }_{31}$ compared to the MLE method. For the sample size 1000, biases on these two parameters increase too, but not so obviously. Variances will also be inflated due to the weights we applied.

**Table 3:** Bias of parameter estimates and MSE from randomly generated data without outliers.

$n$	Parameter	True	MLE			GMWM		
			Bias	MSE	Coverage	Bias	MSE	Coverage
100	${\beta }_{20}$	1.0	0.0666	0.1030	0.945	0.0488	0.1986	0.949
	${\beta }_{30}$	−0.3	−0.0059	0.1206	0.957	−0.1440	0.5578	0.952
	${\beta }_{21}$	−0.8	−0.0654	0.1190	0.938	−0.0513	0.2550	0.961
	${\beta }_{31}$	0.7	0.0566	0.1892	0.963	0.2318	0.5468	0.923
	${\beta }_{22}$	−1.0	−0.0853	0.1764	0.969	−0.0691	0.2380	0.950
	${\beta }_{32}$	−0.5	−0.0624	0.1453	0.945	0.0203	0.3195	0.964
1000	${\beta }_{20}$	1.0	0.0050	0.0087	0.956	0.0043	0.0181	0.962
	${\beta }_{30}$	−0.3	−0.0055	0.0105	0.984	−0.0106	0.0333	0.950
	${\beta }_{21}$	−0.8	−0.0039	0.0099	0.943	−0.0013	0.0251	0.956
	${\beta }_{31}$	0.7	0.0081	0.0160	0.968	0.0162	0.0401	0.954
	${\beta }_{22}$	−1.0	−0.0071	0.0145	0.987	−0.0025	0.0258	0.948
	${\beta }_{32}$	−0.5	−0.0047	0.0122	0.948	0.0041	0.0361	0.947

Outliers are generated from a multivariate normal distribution with the mean vector $=(2,3)$ and identity covariance ${\mathbf{I}}_{2}$. For these outliers, their responses are intentionally misclassified, that is, they are placed within a different category from those predicted categories based on the true parameters.

Table 4 lists simulation results with outliers added. For estimations from datasets with 5% outliers, bias correction from the GMWM is excellent. However, when the datasets have 10% outliers, biases on estimations of some parameters (${\beta }_{21}$ and ${\beta }_{22}$ in this simulation) are decreased, but not completely corrected.

**Table 4:** Comparison between GMWM and MLE estimation from randomly generated data with outliers added.

Size	Parameter	5% contamination						10% contamination					
		GMWM			MLE			GMWM			MLE		
		Bias	MSE	Coverage	Bias	MSE	Coverage	Bias	MSE	Coverage	Bias	MSE	Coverage
100	${\beta }_{20}$	0.0568	0.1102	0.956	0.0860	0.0884	0.957	0.0489	0.0999	0.971	0.0868	0.0819	0.970
	${\beta }_{30}$	−0.0038	0.1427	0.954	−0.0055	0.1528	0.949	−0.0057	0.1510	0.945	−0.0431	0.1461	0.814
	${\beta }_{21}$	−0.0392	0.1464	0.949	0.2377	0.1360	0.785	0.0319	0.1227	0.946	0.3607	0.1933	0.579
	${\beta }_{31}$	0.0175	0.2020	0.944	−0.1072	0.1270	0.921	−0.0235	0.1770	0.943	−0.1631	0.1283	0.949
	${\beta }_{22}$	0.0374	0.1207	0.949	0.3848	0.2115	0.578	0.0207	0.0968	0.945	0.6088	0.4151	0.526
	${\beta }_{32}$	−0.0548	0.1572	0.956	−0.0964	0.0904	0.964	−0.0817	0.1349	0.977	−0.1069	0.0803	0.967
1000	${\beta }_{20}$	0.0172	0.0189	0.939	0.0490	0.0102	0.932	0.0451	0.0202	0.944	0.0657	0.0120	0.900
	${\beta }_{30}$	0.0012	0.0340	0.945	0.0124	0.0075	0.952	−0.0071	0.0336	0.952	−0.0111	0.0063	0.822
	${\beta }_{21}$	0.0260	0.0242	0.937	0.2874	0.0885	0.101	0.0164	0.0207	0.936	0.3876	0.1545	0.002
	${\beta }_{31}$	−0.0058	0.0356	0.950	−0.1423	0.0345	0.697	−0.0497	0.0346	0.917	−0.2269	0.0658	0.521
	${\beta }_{22}$	0.0366	0.0237	0.936	0.4390	0.2032	0.000	0.0238	0.0182	0.938	0.6500	0.4322	0.000
	${\beta }_{32}$	−0.0106	0.0292	0.951	−0.0538	0.0103	0.940	−0.0434	0.0250	0.953	−0.0629	0.0106	0.902

### Application

For the hypertension data, the criterion for identifying observations with large distances is ${c}_{d}=0.22$, and the criterion for identifying leverage points is ${c}_{x}=0.12$. Applying the GMWM estimator, those blue-colored points in Fig. 1 are automatically downweighted, and red-colored points have 0 weight. The GMWM method indeed eliminates those inconsistencies: the coefficient of sodium intake for the odds model between the Stage 2 hypertension and the Normal categories is no longer negative, see the right side of Table 2.

As the results indicate, age, gender, and BMI all had significant impact on hypertension status. For example, one unit increase in BMI resulted in an increase of 1.26 (95% confidence interval [1.16–1.35]) times in likelihood to have Stage 2 hypertension when compared with the normal status. And with one year age increase, a subject was 1.07 (95% CI [1.06–1.10]) times more likely to have Stage 2 hypertension than to stay at the normal healthy status. Contrary to the MLE results for sodium intakes, which were difficult to make a conclusion due to inconsistent estimate, we now find that sodium intakes were statistically significant. When a daily intake of sodium increased one gram, a subject were 1.26 (95% CI [1.15–1.37]) times more likely to have Stage 1 hypertension, and 1.25 (95% CI [1.17–1.35]) times more likely to have Stage 2 hypertension. These results are consistent with the findings from previous studies (National Research Council, 2005; He & MacGregor, 2004).

## Discussion

A reasonable choice to fit ordinal response data is the proportional odds model if the proportional odds assumption is not violated. Proportional odds models can take the ordinal information into modeling. And it reduces the number of parameters which is needed by the generalized logit model. Unfortunately, our data does not met the fundamental assumption of proportional odds models, which makes us choose to treat the outcome as a nominal response.

A datum with a nominal response and some continuous covariates is commonly seen in many scientific areas, such as sociology, economy, and biomedical studies. In order to be able to deal with outliers, we modified the GMM estimator to replace the standard moment conditions with weighted moment conditions, so that aberrant observations automatically receive less weight. We proved that the proposed method has good asymptotic behavior. When outliers are present, the GMWM estimator give much smaller biases than the estimations derived from the traditional MLE method. This method can be adapted to check whether results obtained with the traditional MLE approach are driven only by a few outlying observations. The weights produced from the robust procedure can be used to diagnose the cause of the differences and to indicate routes for model re-specification.

## Appendix: Consistency and asymptotic normality

In this appendix, we introduce the assumptions for the asymptotic analysis of GMWM, and outline the derivations on the main asymptotic properties of GMWM.

We make the following sets of regularity assumptions regarding properties of the moment functions and identification assumptions. Assumption I

I1. $\mathcal{B}$ is a compact parametric space.
I2. $\Sigma $ is a positive definite matrix.
I3. It holds that $E[{u}^{w}(\boldsymbol{\beta})]=0$ if and only if $\boldsymbol{\beta}={\boldsymbol{\beta}}_{0}$, and for any $\epsilon \gt 0$, that
  \begin{eqnarray*} \inf _{\boldsymbol{\beta}\in \mathbf{B}\setminus \mathcal{N}(\boldsymbol{\beta}_{0},\epsilon )}\left\|E[{u}^{w}(\boldsymbol{\beta})]\right\|\gt 0 \end{eqnarray*}
  where $\mathcal{N}({\boldsymbol{\beta}}_{0},\epsilon )=\{ \left.\boldsymbol{\beta}\in {\mathbb{R}}^{l}\right\vert \Vert \boldsymbol{\beta}-{\boldsymbol{\beta}}_{0}\Vert \lt \epsilon \} $ is an open $\epsilon $-neighborhood of a point ${\boldsymbol{\beta}}_{0}$.

Assumption F

F1. Let ${u}^{w}(\boldsymbol{\beta})$ be continuous in $\boldsymbol{\beta}\in \mathcal{B}$, and be twice differentiable in $\boldsymbol{\beta}$ on $\mathcal{N}({\boldsymbol{\beta}}_{0},\epsilon )$ almost surely.
F2. Expectation $E \hspace{0.167em} \sup _{\boldsymbol{\beta}\in \mathcal{B}}\left\|{u}^{w}(\boldsymbol{\beta})\right\|$, $E \hspace{0.167em} \sup _{\boldsymbol{\beta}\in \mathcal{N}(\boldsymbol{\beta}_{0},\epsilon )}\left\|\partial {u}^{w}(\boldsymbol{\beta})/\partial {\boldsymbol{\beta}}_{k}\right\|$, and $E \hspace{0.167em} \sup _{\boldsymbol{\beta}\in \mathcal{N}(\boldsymbol{\beta}_{0},\epsilon )}\left\|{\partial }^{2}{u}^{w}(\boldsymbol{\beta})/\partial {\boldsymbol{\beta}}_{k}\partial {\boldsymbol{\beta}}_{l}\right\|$ exists and are finite for $k,l=1,\ldots ,p.$

Assumption W

W1. $\lim _{\epsilon \rightarrow 0}\sup _{\Vert \Delta \Vert \leq \epsilon }\vert w(\boldsymbol{\beta}+\Delta )-w(\boldsymbol{\beta})\vert =0$.
W2. $\lim _{\epsilon \rightarrow 0}\sup _{\Vert \Delta \Vert \leq \epsilon }\vert \partial w(\boldsymbol{\beta}+\Delta )/\partial \boldsymbol{\beta}-\partial w(\boldsymbol{\beta})/\partial \boldsymbol{\beta}\vert =0$.

 When the above assumptions are met, we can prove that ${\hat {\boldsymbol{\beta}}}_{w}$ is consistent for ${\boldsymbol{\beta}}_{0}$. We begin with studying the behavior of the weighted moment equations in a neighborhood of ${\boldsymbol{\beta}}_{0}$. And proving their asymptotic linearity is followed. The linearity ensures the applicability of the central limit theorem for the asymptotic normality of GMWM. Theorem 1. *Let the assumptions F and I hold, then the GMWM estimator *${\hat {\boldsymbol{\beta}}}_{w}$*is asymptotically normal, that is, *$\sqrt{n}\left({\hat {\boldsymbol{\beta}}}_{w}-{\boldsymbol{\beta}}_{0}\right)\xrightarrow{F}N(0,{M}^{T}{S}^{w}M)$*as *$n\rightarrow \infty $*, where*

\begin{eqnarray*} M=\left(({V}^{w})^{T}\Sigma {V}^{w}\right)^{-1}({V}^{w})^{T}\Sigma , \end{eqnarray*}

\begin{eqnarray*} {S}^{w}=E\left[\frac{1}{n}\sum _{i=1}^{n}{u}^{w}(\hat {\boldsymbol{\beta}}){u}^{w}(\hat {\boldsymbol{\beta}})^{T}\right] \end{eqnarray*}

*with *${V}^{w}=E\left[\frac{\partial {U}^{w}(\hat {\boldsymbol{\beta}})}{\partial {\boldsymbol{\beta}}^{T}}\right]$*.*

We start with proving two lemmas before we present the proof of Theorem 1.

Lemma 1.
*Let the assumptions F, I and W hold, and let* ${U}_{r}^{w}(\boldsymbol{\beta})$ *be the* $r\mathrm{th}$ *element of the vector* ${U}^{w}(\boldsymbol{\beta})$ *,* $r=1,\ldots ,p$ *. Then, for* $0\lt s\lt 1$ *,*

(14) \begin{eqnarray*} \sup _{\Vert t\Vert \leq C}\left\vert \frac{1}{n}\sum _{i}\sum _{l=1}^{p}{t}_{l}\left\{(\partial /\partial {\beta }_{l}){U}_{i,r}^{w}\left(\boldsymbol{\beta}+\frac{s t}{\sqrt{n}}\right)-(\partial /\partial {\beta }_{l}){U}_{i,r}^{w}(\boldsymbol{\beta})\right\}\right\vert ={o}_{p}(1). \end{eqnarray*}

**

Proof. For $l,r=1,\ldots ,p$, by differentiating the $i\mathrm{th}$ component of ${U}_{r}^{w}(\boldsymbol{\beta})$, we get

\begin{eqnarray*} \frac{\partial {U}_{i,r}^{w}(\boldsymbol{\beta})}{\partial {\beta }_{l}}=-w(\boldsymbol{\beta}){X}_{i}\frac{\partial {\pi }_{i}(\boldsymbol{\beta})}{\partial {\boldsymbol{\beta}}_{l}}+\frac{\partial {w}_{i}(\boldsymbol{\beta})}{\partial {\boldsymbol{\beta}}_{l}}{X}_{i}({y}_{i}-{\pi }_{i}(\boldsymbol{\beta})). \end{eqnarray*}

Then,

\begin{eqnarray*} \sup _{\Vert t\Vert \leq C}\left\vert \frac{1}{n}\sum _{i=1}^{n}\sum _{l=1}^{p}{t}_{l}\left\{(\partial /\partial {\beta }_{l}){U}_{i,r}^{w}\left(\boldsymbol{\beta}+\frac{s t}{\sqrt{n}}\right)-(\partial /\partial {\beta }_{l}){U}_{i,r}^{w}(\boldsymbol{\beta})\right\}\right\vert \leq \frac{C}{n}\sum _{i=1}^{n}\sum _{l=1}^{p}{t}_{l}\sup _{\Vert t\Vert \leq C}\left\vert (\partial /\partial {\beta }_{l}){U}_{i,r}^{w}\left(\boldsymbol{\beta}+\frac{s t}{\sqrt{n}}\right)-(\partial /\partial {\beta }_{l}){U}_{i,r}^{w}(\boldsymbol{\beta})\right\vert \end{eqnarray*}

and

\begin{eqnarray*} \sup _{\Vert t\Vert \leq C}\left\vert (\partial /\partial {\beta }_{l}){U}_{i,r}^{w}\left(\boldsymbol{\beta}+\frac{s t}{\sqrt{n}}\right)-(\partial /\partial {\beta }_{l}){U}_{i,r}^{w}(\boldsymbol{\beta})\right\vert \leq \sup _{\Vert t\Vert \leq C}\left\{\left\vert {w}_{i}\left(\boldsymbol{\beta}+\frac{s t}{\sqrt{n}}\right)-{w}_{i}\left(\boldsymbol{\beta}\right)\right\vert \left(\partial /\partial {\beta }_{l}){\pi }_{i}\left(\boldsymbol{\beta}+\frac{s t}{\sqrt{n}}\right)\right\vert \right\}+\sup _{\Vert t\Vert \leq C}\left\{\left\vert (\partial /\partial {\beta }_{l}){\pi }_{i}\left(\boldsymbol{\beta}+\frac{s t}{\sqrt{n}}\right)-(\partial /\partial {\beta }_{l}){\pi }_{i}\left(\boldsymbol{\beta}\right)\right\vert \vert {X}_{i}{w}_{i}\left(\boldsymbol{\beta}\right)\vert \right\}+\sup _{\Vert t\Vert \leq C}\left\{\left\vert (\partial /\partial {\beta }_{l}){w}_{i}\left(\boldsymbol{\beta}+\frac{s t}{\sqrt{n}}\right)-(\partial /\partial {\beta }_{l}){w}_{i}\left(\boldsymbol{\beta}\right)\right\vert \left\vert {X}_{i}\left({y}_{i}-{\pi }_{i}\left(\boldsymbol{\beta}+\frac{s t}{\sqrt{n}}\right)\right)\right\vert \right\}+\sup _{\Vert t\Vert \leq C}\left\{\left\vert \left({y}_{i}-{\pi }_{i}\left(\boldsymbol{\beta}+\frac{s t}{\sqrt{n}}\right)\right)-\left({y}_{i}-{\pi }_{i}\left(\boldsymbol{\beta}\right)\right)\right\vert \left(\partial /\partial {\beta }_{l}){w}_{i}\left(\boldsymbol{\beta}\right)\right\vert \right\}. \end{eqnarray*}

Then, by taking expectation at both sides,

\begin{eqnarray*} E\left\{\sup _{\Vert t\Vert \leq C}\left\vert (\partial /\partial {\beta }_{l}){U}_{i,r}^{w}\left(\boldsymbol{\beta}+\frac{s t}{\sqrt{n}}\right)-(\partial /\partial {\beta }_{l}){U}_{i,r}^{w}(\boldsymbol{\beta})\right\vert \right\}\leq \sup _{\Vert t\Vert \leq C}\left\vert {w}_{i}\left(\boldsymbol{\beta}+\frac{s t}{\sqrt{n}}\right)-{w}_{i}\left(\boldsymbol{\beta}\right)\right\vert \sup _{\Vert t\Vert \leq C}\left(\partial /\partial {\beta }_{l}){\pi }_{i}\left(\boldsymbol{\beta}+\frac{s t}{\sqrt{n}}\right)\right\vert +\sup _{\Vert t\Vert \leq C}\left\vert (\partial /\partial {\beta }_{l}){\pi }_{i}\left(\boldsymbol{\beta}+\frac{s t}{\sqrt{n}}\right)-(\partial /\partial {\beta }_{l}){\pi }_{i}\left(\boldsymbol{\beta}\right)\right\vert \sup _{\Vert t\Vert \leq C}\vert {X}_{i}{w}_{i}\left(\boldsymbol{\beta}\right)\vert +\sup _{\Vert t\Vert \leq C}\left\vert (\partial /\partial {\beta }_{l}){w}_{i}\left(\boldsymbol{\beta}+\frac{s t}{\sqrt{n}}\right)-(\partial /\partial {\beta }_{l}){w}_{i}\left(\boldsymbol{\beta}\right)\right\vert E\left\{\sup _{\Vert t\Vert \leq C}\left\vert {X}_{i}\left({y}_{i}-{\pi }_{i}\left(\boldsymbol{\beta}+\frac{s t}{\sqrt{n}}\right)\right)\right\vert \right\}+\sup _{\Vert t\Vert \leq C}\left\vert \left({y}_{i}-{\pi }_{i}\left(\boldsymbol{\beta}+\frac{s t}{\sqrt{n}}\right)\right)-\left({y}_{i}-{\pi }_{i}\left(\boldsymbol{\beta}\right)\right)\right\vert \sup _{\Vert t\Vert \leq C}\left(\partial /\partial {\beta }_{l}){w}_{i}\left(\boldsymbol{\beta}\right)\right\vert . \end{eqnarray*}

Thus, by conditions F and W, we have

\begin{eqnarray*} E\left\{\sup _{\Vert t\Vert \leq C}\left\vert (\partial /\partial {\beta }_{l}){U}_{i,r}^{w}\left(\boldsymbol{\beta}+\frac{s t}{\sqrt{n}}\right)-(\partial /\partial {\beta }_{l}){U}_{i,r}^{w}(\boldsymbol{\beta})\right\vert \right\}\longrightarrow 0,\quad \forall i \end{eqnarray*}

and

\begin{eqnarray*} E\left\{\sup _{\Vert t\Vert \leq C}\left\vert \frac{1}{n}\sum _{i=1}^{n}\sum _{l=1}^{p}{t}_{l}\left\{(\partial /\partial {\beta }_{l}){U}_{i,r}^{w}\left(\boldsymbol{\beta}+\frac{s t}{\sqrt{n}}\right)-(\partial /\partial {\beta }_{l}){U}_{i,r}^{w}(\boldsymbol{\beta})\right\}\right\vert \right\}\longrightarrow 0,\quad \forall i. \end{eqnarray*}

Therefore, we have the results in (14).∎

Lemma 2.
*Let the assumptions F, I and W hold, it holds that*

(15) \begin{eqnarray*} \frac{1}{\sqrt{n}}\sup _{\Vert t\Vert \leq C}\left\|{U}_{n}^{w}({\boldsymbol{\beta}}_{0}+{n}^{-\frac{1}{2}}t)-{U}_{n}^{w}({\boldsymbol{\beta}}_{0})+{V}^{w}{n}^{-\frac{1}{2}}t\right\|={o}_{p}(1), \end{eqnarray*}

*as* $n\rightarrow \infty $ *, where* ${V}^{w}=E\left[\frac{\partial {U}^{w}(\boldsymbol{\beta})}{\partial {\boldsymbol{\beta}}^{T}}\right]$ *.*

Proof. Write

\begin{eqnarray*} {U}_{n}^{w}({\boldsymbol{\beta}}_{0}+{n}^{-\frac{1}{2}}t)-{U}_{n}^{w}({\boldsymbol{\beta}}_{0})=\sum _{i=1}^{n}{w}_{i}({\boldsymbol{\beta}}_{0}+{n}^{-\frac{1}{2}}t){u}_{i}({\boldsymbol{\beta}}_{0}+{n}^{-\frac{1}{2}}t)-\sum _{i=1}^{n}{w}_{i}({\boldsymbol{\beta}}_{0}){u}_{i}({\boldsymbol{\beta}}_{0}). \end{eqnarray*}

By the Taylor expansion, ${u}_{i}({\boldsymbol{\beta}}_{0}+{n}^{-\frac{1}{2}}t)={u}_{i}({\boldsymbol{\beta}}_{0})+{n}^{-\frac{1}{2}}t\left[\frac{\partial }{\partial \boldsymbol{\beta}}{u}_{i}({\boldsymbol{\beta}}_{0}+\frac{t}{\sqrt{n}})\right]$, where $0\lt s\lt 1$. Then, we can write

(16) \begin{eqnarray*} {U}_{n}^{w}({\boldsymbol{\beta}}_{0}+{n}^{-\frac{1}{2}}t)-{U}_{n}^{w}({\boldsymbol{\beta}}_{0})=\sum _{i=1}^{n}{u}_{i}({\boldsymbol{\beta}}_{0})\left\{{w}_{i}({\boldsymbol{\beta}}_{0}+{n}^{-\frac{1}{2}}t)-{w}_{i}({\boldsymbol{\beta}}_{0})\right\} \end{eqnarray*}

(17) \begin{eqnarray*} +\hspace{0.167em} \frac{1}{\sqrt{n}}\sum _{i=1}^{n}{w}_{i}({\boldsymbol{\beta}}_{0})t\frac{\partial }{\partial \boldsymbol{\beta}}{u}_{i}({\boldsymbol{\beta}}_{0}) \end{eqnarray*}

(18) \begin{eqnarray*} +\hspace{0.167em} \frac{1}{\sqrt{n}}\sum _{i=1}^{n}\left\{{w}_{i}({\boldsymbol{\beta}}_{0}+{n}^{-\frac{1}{2}}t)-{w}_{i}({\boldsymbol{\beta}}_{0})\right\}t\frac{\partial }{\partial \boldsymbol{\beta}}{u}_{i}({\boldsymbol{\beta}}_{0}) \end{eqnarray*}

(19) \begin{eqnarray*} +\hspace{0.167em} \frac{1}{\sqrt{n}}\sum _{i=1}^{n}{w}_{i}\left({\boldsymbol{\beta}}_{0}+\frac{t}{\sqrt{n}}\right)t\left\{\frac{\partial {u}_{i}\left({\boldsymbol{\beta}}_{0}+\frac{t}{\sqrt{n}}\right)}{\partial \boldsymbol{\beta}}-\frac{\partial {u}_{i}({\boldsymbol{\beta}}_{0})}{\partial \boldsymbol{\beta}}\right\}. \end{eqnarray*}

We will now show that terms (16), (18) and (19) are asymptotically negligible. As to the term (16), By assumption W1, $\{ {w}_{i}({\boldsymbol{\beta}}_{0}+{n}^{-\frac{1}{2}}t)-{w}_{i}({\boldsymbol{\beta}}_{0})\} \rightarrow 0$, and ${u}_{i}({\boldsymbol{\beta}}_{0})$ is independent of $\boldsymbol{\beta}$. So we have the term (16) tends to zero. Similarly, $\partial {{/}}\partial \boldsymbol{\beta}{u}_{i}({\boldsymbol{\beta}}_{0})$ is independent of $\boldsymbol{\beta}$ and $t$ is bounded. Hence, the term (18) tends to zero. Lemma 1 implies $\frac{\partial {u}_{i}\left({\boldsymbol{\beta}}_{0}+\frac{t}{\sqrt{n}}\right)}{\partial \boldsymbol{\beta}}-\frac{\partial {u}_{i}({\boldsymbol{\beta}}_{0})}{\partial \boldsymbol{\beta}}\rightarrow 0$, as $n\rightarrow \infty $. So the term (19) can be neglect too. Now, let us analyze the term (17). Let ${w}_{i}^{\ast }({\boldsymbol{\beta}}_{0})$ be the limit of ${w}_{i}({\boldsymbol{\beta}}_{0})$. Rewrite (17) as

(20) \begin{eqnarray*} \frac{1}{\sqrt{n}}\sum _{i=1}^{n}{w}_{i}({\boldsymbol{\beta}}_{0})t\frac{\partial }{\partial \boldsymbol{\beta}}{u}_{i}({\boldsymbol{\beta}}_{0})=\frac{1}{\sqrt{n}}\sum _{i=1}^{n}\left[{w}_{i}({\boldsymbol{\beta}}_{0})-{w}_{i}^{\ast }({\boldsymbol{\beta}}_{0})\right]t\frac{\partial }{\partial \boldsymbol{\beta}}{u}_{i}({\boldsymbol{\beta}}_{0}) \end{eqnarray*}

(21) \begin{eqnarray*} +\frac{1}{\sqrt{n}}\sum _{i=1}^{n}\left\{w_{i}^{\ast }({\boldsymbol{\beta}}_{0})t\frac{\partial }{\partial \boldsymbol{\beta}}{u}_{i}({\boldsymbol{\beta}}_{0})-E\left[{w}_{i}^{\ast }({\boldsymbol{\beta}}_{0})t\frac{\partial }{\partial \boldsymbol{\beta}}{u}_{i}({\boldsymbol{\beta}}_{0})\right]\right\} \end{eqnarray*}

(22) \begin{eqnarray*} +\frac{1}{\sqrt{n}}E\left[{w}_{i}^{\ast }({\boldsymbol{\beta}}_{0})t\frac{\partial }{\partial \boldsymbol{\beta}}{u}_{i}({\boldsymbol{\beta}}_{0})\right]. \end{eqnarray*}

The first term (20) is negligible because $\partial {{/}}\partial \boldsymbol{\beta}{u}_{i}({\boldsymbol{\beta}}_{0})$ is independent of $\boldsymbol{\beta}$, $t$ is bounded, and $\left[{w}_{i}({\boldsymbol{\beta}}_{0})-{w}_{i}^{\ast }({\boldsymbol{\beta}}_{0})\right]\rightarrow 0$. By the central limit theorem, each element of vector (21) converges in distribution to a normally distribution random variable with zero mean and a finite variance which is uniformly bounded by $t$. Hence, (21) is bounded in probability. The last term (20) is

\begin{eqnarray*} \frac{1}{\sqrt{n}}E\left[{w}_{i}^{\ast }({\boldsymbol{\beta}}_{0})t\frac{\partial }{\partial \boldsymbol{\beta}}{u}_{i}({\boldsymbol{\beta}}_{0})\right]=\frac{t}{\sqrt{n}}{V}^{w}. \end{eqnarray*}

This proves the lemma.∎
Proof of Theorem 1. Since ${t}_{n}=\sqrt{n}={\mathcal{O}}_{p}(1)$ as $n\rightarrow \infty $ by Lemma 2, we can write (15) as

(23) \begin{eqnarray*} {U}_{n}^{w}({\boldsymbol{\beta}}_{0}+{n}^{-\frac{1}{2}}{t}_{n})-{U}_{n}^{w}({\boldsymbol{\beta}}_{0})+{V}^{w}{n}^{-\frac{1}{2}}{t}_{n}={o}_{p}({n}^{-\frac{1}{2}}) \end{eqnarray*}

with a probability arbitrarily close to one uniformly in ${t}_{n}\in \{ t:\Vert t\Vert \leq C\} $. Moreover, with ${n}^{-\frac{1}{2}}{t}_{n}={o}_{p}(1)$, $\partial {U}_{n}^{w}({\boldsymbol{\beta}}_{0}+{n}^{-\frac{1}{2}}{t}_{n})/\partial \boldsymbol{\beta}\rightarrow {V}^{w}$ in probability as $n\rightarrow \infty $. Note that the first order conditions of GMWM equal to 0, that is,

\begin{eqnarray*} \frac{\partial {Q}_{n}^{w}({\boldsymbol{\beta}}_{w})}{\partial \boldsymbol{\beta}}={\left[\frac{\partial {U}_{n}^{w}({\boldsymbol{\beta}}_{w})}{\partial \boldsymbol{\beta}}\right]}^{T}\Sigma ({\boldsymbol{\beta}}_{w}){U}_{n}^{w}({\boldsymbol{\beta}}_{w})=0. \end{eqnarray*}

Replace ${U}_{n}^{w}({\boldsymbol{\beta}}_{w})$ with ${U}_{n}^{w}({\boldsymbol{\beta}}_{0}+{n}^{-\frac{1}{2}}{t}_{n})$ from Eq. (23),

\begin{eqnarray*} {\left[\frac{\partial {U}_{n}^{w}({\boldsymbol{\beta}}_{0}+{n}^{-\frac{1}{2}}{t}_{n})}{\partial \boldsymbol{\beta}}\right]}^{T}\Sigma ({\boldsymbol{\beta}}_{w}){U}_{n}^{w}({\boldsymbol{\beta}}_{0}+{n}^{-\frac{1}{2}}{t}_{n})={\left[{V}^{w}+{o}_{p}(1)\right]}^{T}\Sigma ({\boldsymbol{\beta}}_{w})\left[{U}_{n}^{w}({\boldsymbol{\beta}}_{0})-{V}^{w}{n}^{-\frac{1}{2}}{t}_{n}\right]=0. \end{eqnarray*}

Then we have

(24) \begin{eqnarray*} {t}_{n}=\sqrt{n}({\boldsymbol{\beta}}_{w}-{\boldsymbol{\beta}}_{0})=\sqrt{n}\left[({V}^{w})^{T}\Sigma ({\boldsymbol{\beta}}_{w}){V}^{w}\right]^{-1}({V}^{w})^{T}\Sigma ({\boldsymbol{\beta}}_{w}){U}_{n}^{w}({\boldsymbol{\beta}}_{0})+{o}_{p}(1). \end{eqnarray*}

Next we examine the behavior of $\sqrt{n}{U}_{n}^{w}({\boldsymbol{\beta}}_{0})$, which can be written as

(25) \begin{eqnarray*} \sqrt{n}{U}_{n}^{w}({\boldsymbol{\beta}}_{0})={n}^{-\frac{1}{2}}\sum _{i=1}^{n}{u}_{i}({\boldsymbol{\beta}}_{0}){w}_{i}({\boldsymbol{\beta}}_{0})={n}^{-\frac{1}{2}}\sum _{i=1}^{n}{u}_{i}({\boldsymbol{\beta}}_{0})\left\{{w}_{i}({\boldsymbol{\beta}}_{0})-{w}_{i}^{\ast }({\boldsymbol{\beta}}_{0})\right\} \end{eqnarray*}

(26) \begin{eqnarray*} +\hspace{0.167em} {n}^{-\frac{1}{2}}\sum _{i=1}^{n}{u}_{i}({\boldsymbol{\beta}}_{0}){w}_{i}^{\ast }({\boldsymbol{\beta}}_{0}). \end{eqnarray*}

Note that the term (25) is asymptotically negligible in probability due to the triangle inequality and assumption W1. The term (26) is a stationary sequence of absolutely random variables. By assumption I3 and F2, (26) have zero mean and finite second moments. So the central limit theorem can be applied on (26), giving $\sqrt{n}{U}_{n}^{w}({\boldsymbol{\beta}}_{0})\sim N(0,{S}^{w})$ (Davidson, 1994, Section 25.3) With Eq. (24), we have asymptotic normality of ${\boldsymbol{\beta}}^{w}$, and its asymptotic variance is given by ${M}^{T}{S}^{w}M$ (Davidson, 1994).∎
